# Histological staining methods preparatory to laser capture microdissection significantly affect the integrity of the cellular RNA

**DOI:** 10.1186/1471-2164-7-97

**Published:** 2006-04-27

**Authors:** Hongyang Wang, James D Owens, Joanna H Shih, Ming-Chung Li, Robert F Bonner, J Frederic Mushinski

**Affiliations:** 1 Laboratory of Genetics, Center for Cancer Research, National Cancer Institute, NIH, Bethesda, MD 20892, USA; 2 Biometric Research Branch, Division of Cancer Treatment and Diagnosis, National Cancer Institute, NIH, Bethesda, MD 20892, USA; 3 The Emmes Corporation, Rockville, MD 20850, USA; 4 Laboratory of Integrative and Medical Biophysics, National Institute of Child Health and Human Development, NIH, Bethesda, MD 20892, USA; 5 Division of Rheumatology and Immunology, Department of Internal Medicine, University of Virginia, Charlottesville, VA 22906, USA; 6 Bldg. 37, Room 3134C, NIH, 37 Convent Drive, MSC-4258, Bethesda, MD 20892-4258, USA

## Abstract

**Background:**

Gene expression profiling by microarray analysis of cells enriched by laser capture microdissection (LCM) faces several technical challenges. Frozen sections yield higher quality RNA than paraffin-imbedded sections, but even with frozen sections, the staining methods used for histological identification of cells of interest could still damage the mRNA in the cells. To study the contribution of staining methods to degradation of results from gene expression profiling of LCM samples, we subjected pellets of the mouse plasma cell tumor cell line TEPC 1165 to direct RNA extraction and to parallel frozen sectioning for LCM and subsequent RNA extraction. We used microarray hybridization analysis to compare gene expression profiles of RNA from cell pellets with gene expression profiles of RNA from frozen sections that had been stained with hematoxylin and eosin (H&E), Nissl Stain (NS), and for immunofluorescence (IF) as well as with the plasma cell-revealing methyl green pyronin (MGP) stain. All RNAs were amplified with two rounds of T7-based in vitro transcription and analyzed by two-color expression analysis on 10-K cDNA microarrays.

**Results:**

The MGP-stained samples showed the least introduction of mRNA loss, followed by H&E and immunofluorescence. Nissl staining was significantly more detrimental to gene expression profiles, presumably owing to an aqueous step in which RNA may have been damaged by endogenous or exogenous RNAases.

**Conclusion:**

RNA damage can occur during the staining steps preparatory to laser capture microdissection, with the consequence of loss of representation of certain genes in microarray hybridization analysis. Inclusion of RNAase inhibitor in aqueous staining solutions appears to be important in protecting RNA from loss of gene transcripts.

## Background

**Figure 1:**
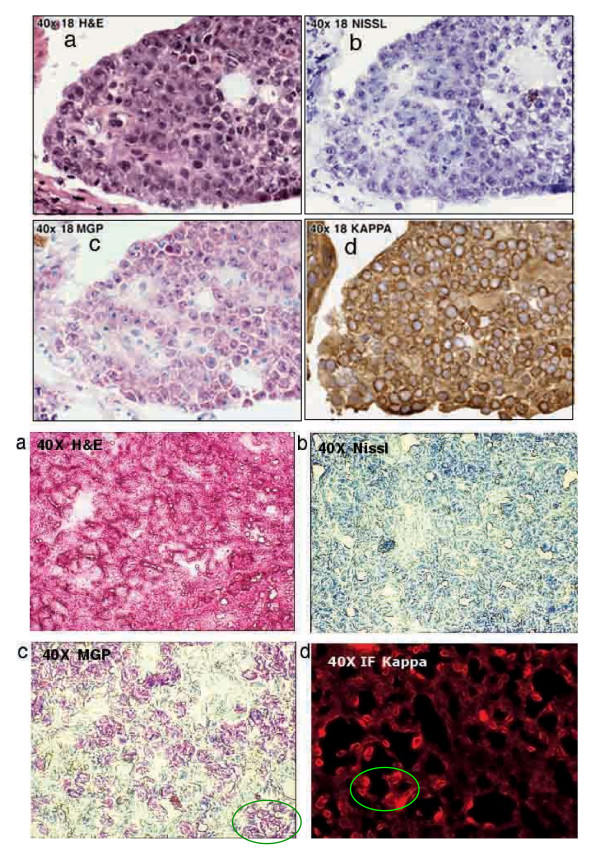
Histochemical staining of frozen sections of an intraperitoneal primary mouse plasma cell tumor. A nodule rich in plasma cells can be seen in the midst of surrounding stromal and fat cells. The stains of the sections are as follows: H&E, hematoxylin and eosin; Nissl; MGP (methyl green pyronin) and kappa (immunoperoxidase in the top four panels or immunofluorescence in the bottom four panels) staining of immunoglobulin kappa light chains). The images in the top four panels represent frozen sections stained and mounted under cover slips in a distant histology laboratory and captured using high quality optics in a photographic studio. Those in the bottom four panels reflect the challenges of interpreting microscopic views on a microscope optimized for LCM, viewing frozen sections that had been processed and stained with the utmost speed to minimize RNA damage and lacking cover slips. Ovals indicate clusters of plasma cells with characteristic MGP staining and rich in Ig κ protein.

Microarray hybridization has been used to study the global gene expression from many different kinds of tissues and cell lines [1-4]. When it is desired to apply this technique only to certain cells that exist in a heterogeneous tissue, surrounded by cells of other types such as connective tissue cells, it is essential to minimize the contribution of mRNA from undesirable cells by enriching the percentage of desirable cell types [5]. Laser Capture Microdissection (LCM) is a valuable tool that makes this possible via the visual (microscopic) identification of cells of interest in intact tissues, followed by their excision and subsequent RNA extraction and analysis by microarray hybridization analysis [6-8]. Frozen sections are highly recommended to maximize quantity and quality of RNA recovery [9,10]. However, in frozen sections it is often difficult to recognize histological details after routine staining, such as hematoxylin & eosin (H&E), in part because LCM requires desiccated sections with no cover slip. Specialized staining methods may be helpful for distinguishing cells of interest from surrounding stroma, e.g., Nissl stain (NS), Immunofluorescence (IF), and Immunohistochemistry (IHC) [7,11,12], but these reagents potentially could result in RNA damage. Methyl Green Pyronin (MGP) is a special stain that has been useful in identifying plasma cells, a major interest of this laboratory, owing to its staining of the nucleus dark blue and the cytoplasm bright pink in visible light or fluorescing red under UV illumination of paraffin-imbedded sections [13]. Although MGP and all histological stains yield significantly less detail on frozen sections than on paraffin-embedded tissue, MGP did enable identification of plasma cells in the midst of other cell types in oil granulomas (Figure 1B, panel c). Since the pyronin reagent reacts with RNA, we worried that MGP might damage the RNA and compromise the subsequent gene expression profiling. Therefore, we investigated whether MGP or other commonly used staining methods themselves would affect gene expression profiling and to what extent.

Usually, a minimum of 5–50 μg total RNA is required for indirect or direct labeling of cDNA, respectively, with fluorochromes such as Cy3 and Cy5 to perform hybridization on one microarray chip to analyze gene expression [14]. RNA yields of this magnitude are usually impossible from the small number of cells that typically comprise LCM-derived samples, requiring amplification to make cDNA microarray analysis possible. RNA amplification by RNA polymerase-based *in vitro *transcription is thought to introduce the least bias to gene expression profiling [15-17], although it is possible that amplifications methods have the potential of amplifying the effect of damage to mRNAs introduced during LCM processing.

It has not been determined to what extent each of the steps in LCM sample processing damages the RNA, potentially leading to loss of representation of certain genes when amplified, reverse transcribed, labeled and hybridized to a microarray containing a wide spectrum of cDNA targets on a glass slide. The purpose of the present study was to assess how much the choice of histological staining before LCM would contribute to gene loss, recognized as dropout of hybridized spots, when compared with unstained samples. To render our analysis statistically significant we performed four biological replicates for each staining protocol, starting with 16 individual LCM-derived tissue samples. Each of these 16 was compared with a pool of RNA from an identical pellet of cells. We found that the MGP staining method, which included RNAase inhibitor, was the least detrimental to the RNA, while the Nissl method, which had no RNAase inhibitor, inflicted the most damage to the RNA.

## Results

**Figure 2:**
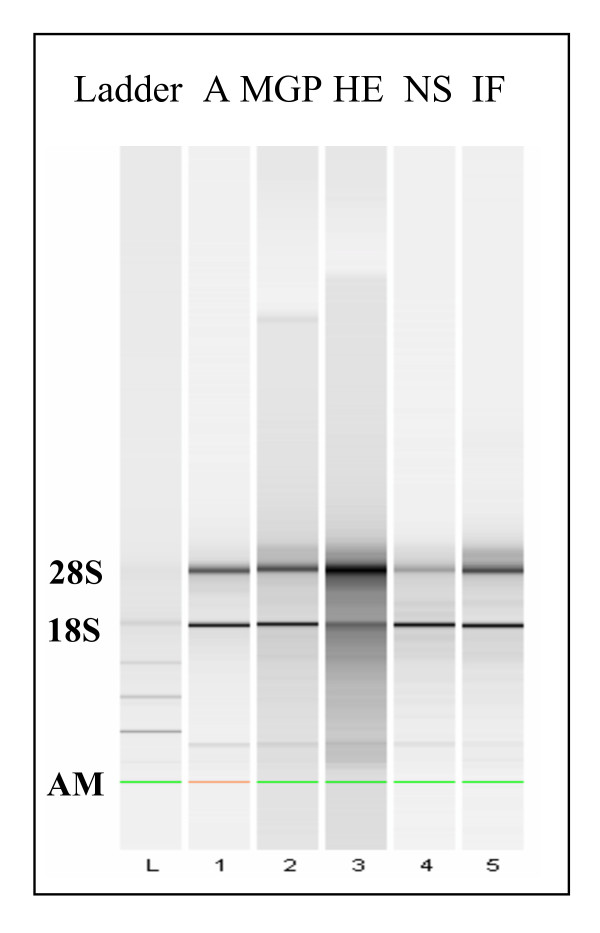
Analysis of RNA quality. One ng of total RNA from one sample from each staining group was electrophoresed on Agilent Bioanalyzer system (Pico RNA chip). RNA ladder 6000 (Ambion, Inc). Lane A contains total RNA from unsectioned cell pellets. Lanes marked MGP, H&E, NS and IF indicate the staining method used before LCM procurement and RNA preparation. Lowermost band (green) in all lanes is an Agilent alignment marker.

RNA was extracted from pellets of cultured TEPC 1165 murine plasma cell tumor cells, which had been flash frozen immediately after harvest. LCM-derived RNA was isolated from samples of microdissected frozen sections of an identical cell pellet, which had been treated with different staining methods. To obtain a sufficient amount of total RNA from LCM-captured cells, we procured cells with 2000–2500 hits of laser (approximately 4000–5000 cells in total). All LCM-captured samples yielded sufficient total RNA (10 – 20 μg) that could be quantitated by the low-range Ribogreen RNA Quantitation Kit (Molecular Probes). Each RNA sample was evaluated for suitability for use by electrophoresis using Agilent RNA Pico chips. RNA that was not degraded should have two clear bands representing the 28S and 18S rRNA, with ratios of 28S/18S absorbance exceeding 1.5. In addition, the RNA sample should be low in genomic DNA contamination, which would appear as bands larger than 28S rRNA. RNA from the unprocessed cell pellet had an acceptable 28S/18S rRNA ratio of about 1.5. The four LCM samples also showed clear 28 rRNA and 18 rRNA bands, but the 28S/18S ratios were different: 1.75 for the unprocessed cell pellet (A), 1.84 for MGP, 4.76 for H&E, 0.71 for NISSL, 2.05 for IF, indicating that a small amount of degradation appeared in the Nissl RNAs (Figure 2) the only sample with a ratio less than 1.7. The unusually high ratio on the H&E lane appears to stem from the unusual and not yet understood high background, since substantial amounts of 28S are clearly visible to the naked eye. Five ng of total RNA from all samples were subjected to 2 rounds of amplification. All samples yielded sufficient amplified antisense RNA for microarray hybridization. After two rounds of amplification the total yield of antisense RNA from all samples ranged from 40 μg to 60 μg. The size of the antisense RNA after 2 rounds of amplification was in the expected and satisfactory range between 200 NT to 700 NT for all the samples (data not shown), indicating that these antisense RNAs could be used directly for cDNA synthesis and probe labeling for microarray hybridization. We did not perceive any consistent difference in yield or size of amplified RNAs that could be attributed to the method of staining.

### Comparisons between LCM subgroups

**Figure 3:**
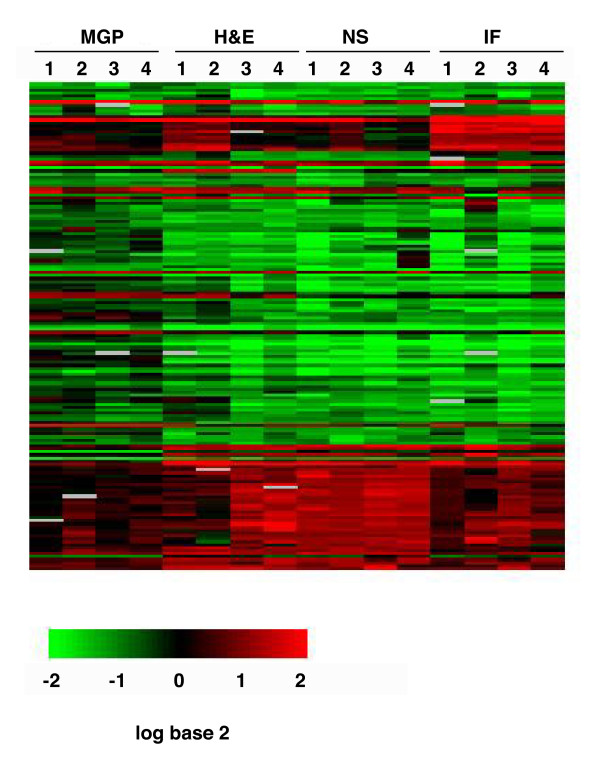
Log-ratios of genes significantly different between A (cell pellet) and LCM samples where genes are sorted according to the order of a dendrogram generated by clustering over genes. Red shades correspond to higher expression in the staining group than A; green shades correspond to lower expression; and black indicates no difference.

We found significant differences between the molecular profiles of each "LCM + staining" group and the unprocessed cell pellet (A). One-sample t-tests identified 74, 76, 108, and 77 genes differently expressed at the 0.001 significance level between the unprocessed cell pellet and MGP, H&E, NS and IF, respectively. Testing 8,534 probes at this significance level, we expected that the average number of spuriously significant (false positive) results would be nine or less. Thus, the expression profiles in the staining groups are significantly different from those of the unprocessed cell pellet. Among these genes, 21, 44, 70, and 44 had at least 2-fold mean expression difference for MGP, H&E, NS and IF, respectively (Table 1) of which 13, 29, 40, and 31 had lower expression. The heat map for the union of the genes differentially expressed (p < 0.001 and ≥ 2-fold mean expression difference) between each LCM + staining sample and the pool of cell pellet preparations (Figure 3) confirmed graphically that the MGP staining method introduced the fewest differences. The MGP vs. A comparison in the 4 left-most columns showed the largest number of black elements that indicate neither loss nor gain of signal following staining.

In addition, we tested the difference in expression profiles among the H&E, NS, IF samples and the MGP samples (Table 2). The number of genes significant at the 0.001 level and with a mean expression difference greater than 2-fold is equal to 61, 147, and 112 for H&E, NS and IF, respectively. Most of these genes (52, 115, and 89 for H&E, NS and IF, respectively) had weaker signals in these three groups of samples, compared with the MGP-stained group, suggesting loss of signal at additional spots, owing to RNA damage acquired during staining. The global permutation tests also showed that the overall signals in the H&E, NS and IF samples were significantly weaker than in the MGP samples (p-values <0.02).

Loss of mRNA suitable for hybridizing to some of the cDNAs was the result expected from damage during processing, and this loss was expected to show no gene specificity. The accuracy of this prediction can be visually appreciated in the heat maps presented in Figure 3. Thus, no additional analysis was done to analyze which specific genes were involved in expression changes among the individual chips.

Superimposed upon this experimentally induced spot drop out are the differences inherent in the microarray hybridization method. The number of spots that show increased intensities (always lower than the number of spots with decreased intensities) may be a rough measure of this effect in each subgroup. Thus, we subtracted this value form the number of spots with lower intensities in the stained samples to yield a value that can be used for inter-group comparison.

## Discussion

The cDNA microarray hybridization method is now widely used to analyze simultaneously the expression of thousands of genes in cancer tissues [5,21], but the contamination of tumors by epithelial, stromal or immune cells presents problems for obtaining accurate expression profiles. Microarray analysis coupled with LCM is a good way to solve this problem. However, cDNA microarray analysis requires a large amount of total RNA (5 μg to 100 μg). It has been shown that one or two rounds of amplification can be used reliably, without compromising RNA quality or gravely skewing patterns of gene expression, when only small initial amounts of total RNA are available [16,17,22]. In this study, we used two rounds of amplification to obtain amounts of antisense RNA sufficient for microarray hybridization yielding strong signal intensities, permitting statistical data processing.

To positively identify the specific cells desired for the microdissection, a staining protocol must be used, which permits identification of plasma cells in desiccated cryosections. Such sections, without cover slips, are required for microdissection and may pose a critical problem, since desiccate cryosections are not optically ideal for histological examination (cf. Figure 1). However, staining solutions may react with cellular components, such as RNA, potentially adversely affecting RNA integrity. In addition, the aqueous components of the staining reagents may contain or activate intracellular RNAases and result in RNA degradation. For this reason, it was important to test different conditions for tissue staining and section dehydration to assure high quality of RNA from LCM samples. To assure ourselves of adequate numbers of uniform populations of plasma cells, which are scarce in most tissues, we utilized pellets of cultured plasma cell tumor cells that were sectioned and stained as if they had been collected by LCM. We used this approach, because obtaining large enough sample of plasma cell tumor cells from tissues by LCM would have been impracticable.

Our modification (addition of RNAase inhibitor) to the standard MGP staining method has made it suitable for use with LCM. Sections treated as described here can be used to prepare total RNA of good quality. Though pyronin is a fluorescent molecule that can bind to RNA, we detected only 21 changes when compared with unstained LCM samples. This amounted to less than 0.3% of the analyzed microarray elements, and less than one half of the number changed due to the other staining methods tested.

It appears that inclusion of RNAase inhibitor in staining solutions can prevent RNA from damage by endogenous and exogenous RNAase. The Nissl-stained group was the only staining protocol that did not include RNAase inhibitor. The number of genes in this group with 2-fold changes, 70 spots, was the highest among all four groups, indicating that this staining procedure damaged the most mRNAs.

In summary, we performed a statistical analysis of 16 cDNA microarray chips comparing fluorescent-labeled cDNA generated from amplified RNA from 16 independently isolated and processed, LCM-procured, stained frozen sections with amplified RNA from an identical pellet of plasma cell tumor cells. This showed that a variable amount of spot fall-out was seen after staining of the frozen sections, depending on the staining method, indicating that the choice of staining method can be important in minimizing loss of mRNAs from microarray analysis of LCM-derived RNA. Happily, the degree of mRNA damage was small with the commonly used H&E method, so long as RNAase inhibitor was included in the aqueous hematoxylin solution. Nissl staining solution, which may not be compatible with RNAase inhibitor addition, introduced the greatest degree of damage. Fortunately, for our ongoing study of plasma cell tumor development, the MGP stain, chosen for its unique ability to flag plasma cells in tissue sections, was the least destructive of all. Our analysis suggests that maximizing the use of non-aqueous staining solutions is recommended for other specialty stains that might be advantageous for identification of other cell types in studies that require use of LCM. The inclusion of RNAase inhibitor in aqueous solutions appears to be important in rendering histological staining procedures suitable to extraction of intact RNA.

## Conclusion

RNA damage can occur during the staining steps preparatory to laser capture microdissection, resulting in loss of representation of certain genes in microarray hybridization analysis. Inclusion of RNAase inhibitor in aqueous staining solutions appears to be important in protecting RNA from loss of gene transcripts.

## Methods

### Cell culture

The murine plasma cell tumor cell line TEPC 1165 [18] was used both as a source of frozen sections for LCM as well as a source of reference RNA, i.e., RNA extracted without LCM processing. Cells were grown in RPMI 1640 media supplemented with 10% fetal bovine serum, 10 ng/ml IL-6, 4 mM L-glutamine and 100 units/ml penicillin and 100 units/ml streptomycin. Duplicate cell pellets were collected by low-speed centrifugation in 50-ml conical centrifuge tubes and immediately snap frozen for RNA extraction and LCM preparation.

### Preparation of LCM samples

The cell pellets were frozen in a dry ice-ethanol bath, embedded with OCT frozen tissue embedding medium, and stored at -80°C. Six-micrometer serial frozen sections were cut on a cryostat at -20°C and mounted on chilled, pre-cleaned, uncoated microscope glass slides that had been heated at 200°C overnight to eliminate RNAase. Slides were kept on dry ice or at -80°C until LCM was completed. Sections were stained individually with four different methods before LCM. RNAase-free (treated with diethylpyrocarbonate) water was used for all solutions. All procedures were carried out at room temperature, unless otherwise stated, and usually on the same day as the frozen sectioning.

#### Methyl Green Pyronin staining

After fixation in 70% ethanol (10 sec), each slide was rinsed in DEPC-treated water (5 sec). Then, 50 μl of MGP staining solution (Sigma/Aldrich) to which RNAase inhibitor (SUPERase*In, Ambion) had been added to a final concentration of 500 U/ml, was applied to the section and incubated 2 min, followed by a quick dip in diethylpyrocarbonate-treated water, dehydration in 100% ethanol in 2 steps (quick dip, then for 10–15 sec), and xylenes in 2 steps (2 min then 3 min). It is important to note that MGP, like all histological stains, yields significantly less detail on frozen sections than on paraffin-embedded tissue, in part, owing to lack of a cover slip. MGP did enable identification of plasma cells in oil granulomas under these conditions. To be used in this way, however, one must include RNAase inhibitor if RNA extraction is desired after LCM.

#### Hematoxylin and eosin staining

A modification was incorporated into the manufacturer's (Sigma/Aldrich) protocol; namely, RNAase inhibitor (SUPERase*In, Ambion) was added to the aqueous hematoxylin solution to a final concentration of 500 U/ml. Briefly, slides were stained as follows: 70% EtOH for 10 sec, DEPC-treated water for 5 sec, hematoxylin with RNAase inhibitor for 20 sec, 70% EtOH for 30 sec, eosin Y in 100% EtOH for 20 sec, followed by dehydration with a series of alcohol for 30 sec each, and xylenes for 2 min.

#### Nissl staining

The frozen sections were fixed with 70% ethanol (30 sec), rinsed in DEPC-treated water (30 sec), immersed in NS staining solution (Arcturus Corp.) for 30 sec, dehydrated with graded alcohols (30 sec each) and xylenes (2 min and 3 min).

#### Immunofluorescence

Sections were fixed in ice-cold acetone for 2 min, rinsed in DEPC-treated PBS for 10 sec, incubated with a 1:250 dilution of goat anti-mouse Igκ conjugated with TXRD (Southern Biotechnology Associates, Inc.) to which RNAase inhibitor (SUPERase*In, Ambion) had been added to a final concentration of 500 U/ml, for 2 min in the dark, dehydrated with 100% ethanol and xylenes (11).

### Laser capture microscopy

Cells were lifted from the slide onto CapSure LCM plastic caps (Arcturus Corp.) using a 30-μm spot size of laser at the power of 50 μv using PixCell II LCM system (Arcturus Corp.).

### RNA extraction and purification

Total RNA from the unprocessed cell pellet was extracted using TriZol reagent (Sigma), treated with RNAase-free DNAase I (Qiagen, Inc.), followed by RNA purification with Qiagen RNeasy kit according to the manufacturer's protocol. RNA concentration was measured spectrophotometrically.

RNA extraction of 16 LCM-derived samples (4 from each staining group) was performed with the PicoPure RNA extraction kit (Arcturus Corp.), simultaneously treated with RNAase-free DNAase I (Qiagen, Inc) according to the manufacturer's protocol. Briefly, 50 μl of extraction buffer was pipetted onto the tissue fragments that had been collected in the cap of a sterile plastic 1.5-micro-centrifuge tube (CapSure LCM caps, Arcturus Corp.), and they were incubated together in a hot-air oven at 42°C for 30 min. Cells and buffer were collected by centrifugation at 800 × g for 2 min. RNA was DNAase-treated and purified on a preconditioned RNA-purification spin column according to the manufacturer's recommendations. LCM-derived total RNA was quantitated with Ribogreen (Molecular Probes, Inc.) using Victor 2 1420 multilabel counter (Perkin-Elmer/Wallac). To assess the RNA quality and confirm the absence of genomic DNA contamination, the Agilent 2100 Bioanalyzer (Pico RNA chip) was used to perform electrophoresis on picogram amounts of total RNA. Only samples with intact 18S and 28S rRNA peaks were used for gene expression analysis.

### RNA amplification

We performed antisense RNA amplification of each RNA sample with RiboAmp™ RNA Amplification kit (Arcturus, Inc.) based on T7 RNA polymerase *in vitro *transcription of cDNA synthesized by reverse transcription primed by oligo-dT attached to the T7 RNA polymerase promoter [15]. To avoid bias caused by different starting amounts of total RNA in the amplification process, 5 ng of total RNA were used in all experimental and reference samples. To obtain sufficient antisense RNA for microarray experiments, two rounds of amplification were performed on all samples. The total yield of amplification products was determined using Ribogreen (Molecular Probes, Inc.), and the sizes of the amplified RNAs were determined with the Agilent Bioanalyzer (nano RNA chip).

### Probe labeling and cDNA array hybridization

Probes were synthesized and labeled from 4 μg of amplified RNA (aRNA) by the method of Xiang et al [14]. In brief, 4 μg of amplified RNA were combined with 4 μg amine-modified random primer and 5 units of RNAase inhibitor (SUPERase*In, Ambion). The mixture was incubated at 70°C for 10 min, then chilled on ice for 10 min, and left at room temperature for 10 min. Primer-RNA solution was added to the reverse transcriptase mix (including 0.5 mM dATP, dGTP, dCTP, 0.3 mM dTTP, and 0.2 mM aminoallyl-dUTP) and incubated at 42°C for 2 h. The reaction was terminated by adding 10 μl 0.5 M EDTA, and RNA was hydrolyzed with 10 μl 1 M NaOH at 65°C for 30 min. The solution was neutralized with 10 μl 1 M HCl, and then MinElute PCR purification kits (Qiagen, Inc) were used to purify the cDNAs. The cDNA was eluted three times with 10 μl of H_2_O. In each staining group 3 of the 4 reference RNA samples were labeled with Cy5, while 1 of the 4 was labeled with Cy3. Three of each group of 4 experimental samples were labeled with Cy3, and 1 of 4 was labeled with Cy5 to test for bias from labeling. Probes were mixed and hybridized overnight at 42°C in hybridization solution (New England Nuclear) to microarray chips with a total of 9998 cDNA elements (InCyte) printed by the NCI microarray facility.

### Evaluation of gene-specific dye bias

Four arrays were hybridized for each of the 4 groups (MGP, HE, IF, NS). In each group 3 arrays had cDNA derived from the reference, unsectioned pellet of cells labeled with Cy5 and the LCM-derived test sample labeled with Cy3, and one array had the labeling reversed. We were interested in evaluating the possibility of gene-specific dye bias that was not removed by the normalization process. To evaluate this possibility, we computed the average difference in log2 ratio between normalized forward- and reverse-label experiments for each array element as an estimate of the residual dye bias for the associated gene. Of the 8534 array elements, none had an average difference of log2 ratio in absolute value greater than 1 (i.e., 2-fold difference). Thus, we concluded that after normalization there was no systematic bias favoring either dye.

### Array scanning and data processing

Arrays were scanned at 10-μm resolution using a GenePix 4000 (Axon Inc.) array scanner. PMT voltages were varied to gain optimum intensity of the spots. Four duplicate hybridizations/slides were used in each experiment. LCM-derived RNAs were regarded as "experimental" samples, and 3 of the 4 cDNA probes derived from them were labeled with Cy3, whereas the "reference" samples, derived from RNA from unprocessed cell pellets were usually labeled with Cy5. To rule out color bias produced by the labeling process itself, the fluorochromes were reversed once in each group of four repeats. Quantification files were obtained by using the GenePixPro 4.0 software (Axon Inc.). The fluorescence signal intensity was determined as the volume in a fixed-size circle, and background was estimated as the median pixel value in a diamond-shaped region between each adjacent four-spot region.

### Data filtering and normalization

Log-ratios of local median background-subtracted intensity levels were analyzed. For each array element, if the intensity was less than 75 in one channel but greater than 75 in the other channel, the intensity <75 was set at 75. Array elements that were flagged as poor quality during image analysis or that had intensities less than 75 in both channels were not reliable and treated as missing [19]. Six percent of the array elements were found to be missing in this way. Those samples with missing values for a particular array element were excluded from all analyses involving that array element. In order to have enough samples in each group to compare expression profiles between the groups, array elements were further filtered out and removed from analysis if their number of non-missing values in each group was less than three. Eleven percent of the array elements were filtered out in this way, resulting in 8534 array elements for analysis. Log-ratios for each microarray were normalized by the locally robust lowess smoother (20) to adjust for any dye bias and variation in PMT voltage settings.

### Clustering

Average linkage hierarchical analysis of differentially expressed genes was performed by using 1-Pearson correlation as the distance metric to group genes based on their patterns of variation across the staining groups. The clusters and associated heat maps were implemented in R.

### Enumeration of differentially expressed genes

Identification of array elements that were differentially expressed between each subgroup and the reference sample and among subgroups was done using one-sample t-tests and two-sample t-tests, respectively. Differentially expressed genes were identified as those genes that were significant at the 0.001 level (p < 0.001) and which were at least 2-fold different in the geometric mean of the expression measurements. A treated subgroup was considered to have weaker expression than another treated subgroup if, among the genes that were differentially expressed between these two subgroups, it had more lower-expressed genes than higher-expressed genes. A global permutation test was used as a global test to assess if one subgroup had statistically significant weaker expression than the other subgroup. Specifically, two-sample t-tests were performed by randomly permuting the group labels. In each permutation, the difference between the number of lower-expressed genes and the number of higher-expressed genes was recorded. The p-value of the permutation test was the proportion of permutations with the same or larger difference as those observed in the original data. Two subgroups would be defined as having globally significant different expression profiles if the p-value from the permutation test is less than 0.05.

## Abbreviations

LCM (Laser Capture Microdissection), MGP (Methyl Green Pyronin), H&E (Hematoxylin and Eosin), NS (Nissl Stain), IF (Immunofluorescence).

## Authors' contributions

HW, RFB and JFM conceived the idea for the project. HW and RFB performed the laser capture microdissection. JDO prepared and quality controlled some of the RNAs. HW performed the microarray hybridizations and scans. JHS and M-CL performed the statistical analysis and interpretation.

## Figures and Tables

**Table 1:** Number of genes significantly differently expressed (p < 0.001 and > 2-fold mean expression difference) after LCM and staining.

**Intensity Ratios**	**MGP/A**	**H&E/A**	**NS/A**	**IF/A**
Total number	21	44	70	44
2-fold lower	13	29	40	31
2-fold higher	8	15	30	13
Net lower	5	14	10	18

Shown are numbers of genes with greater than two-fold change in expression when means of each group, e.g., MGP-stained, LCM-procured cells, were compared with the gene expression profile "A" from the pelleted, unstained sample (not LCM-procured). Net genes lower was calculated as total genes lower minus the number higher.

**Table 2:** Number of spots differently hybridizing after staining with stains other than MGP.

**Intensity Ratios**	**H&E/MGP**	**NS/MGP**	**IF/MGP**
Total number	61	147	112
2-fold lower	52	115	89
2-fold higher	9	32	23
Net lower	43	83	66

Shown are numbers of genes with greater than two-fold change in expression when means of each group, e.g., H&E-stained, LCM-procured cells, were compared with the gene expression profile of the MGP-stained, LCM-procured RNA. Net genes lower was calculated as total genes lower minus the number higher.
